# High Expression of Citron Kinase Contributes to the Development of Esophageal Squamous Cell Carcinoma

**DOI:** 10.3389/fgene.2021.628547

**Published:** 2021-07-07

**Authors:** Wenfeng Lu, Yun Dong, Qing Cui, Yuhan Wang, Xiwen Yang, Xiaoyue Cai, Ming Zhang

**Affiliations:** ^1^Department of Integrative Medicine, Zhongshan Hospital and Laboratory of Neurology, Institute of Integrative Medicine, Fudan University, Shanghai, China; ^2^Department of Integrative Medicine, Shanghai Chest Hospital, Shanghai Jiao Tong University, Shanghai, China

**Keywords:** esophageal squamous cell carcinoma, citron kinase, cell proliferation, apoptosis, tumorigenicity

## Abstract

**Objective:**

This study aimed to investigate the role and potential regulatory mechanism of citron kinase (CIT) in esophageal squamous cell carcinoma (ESCC).

**Methods:**

Citron kinase (CIT) expression in ESCC tissues was analyzed based on the microarray dataset GSE20347, and CIT expression in ESCC cell lines was analyzed. Eca-109 cells were lentivirally transfected with shRNA-CIT (LV-shCIT) to knock down CIT, followed by investigation of cell proliferation and apoptosis. Nude mouse xenograft experiments were performed to evaluate the tumorigenicity of CIT-knockdown Eca-109 cells. Microarray analysis of Eca-109 cells transfected with LV-shCIT or LV-shNC and subsequent Ingenuity Pathway Analysis (IPA) were performed to identify CIT-related differentially expressed genes (DEGs) and signaling pathways. Furthermore, the expression of key DEGs was validated using the clinical samples of ESCC.

**Results:**

Citron kinase (CIT) was highly expressed in ESCC tissues and cell lines. Knockdown of CIT suppressed Eca-109 cell proliferation and promoted apoptosis *in vitro*. Moreover, CIT knockdown significantly reduced tumorigenicity of Eca-109 cells *in vivo*. Microarray and IPA analysis showed that signaling by the Rho family GTPases pathway was significantly activated, and CIT intrinsically interacted with the protein kinase AMP-activated catalytic subunit alpha 1 (PRKAA1), sequestosome 1 (SQSTM1), and interleukin 6 (IL6). Notably, the expression levels of PRKAA1 and SQSTM1 were upregulated in ESCC tissues, while the IL6 expression was downregulated.

**Conclusion:**

Our findings confirm that CIT functions as an oncogene in ESCC. CIT may contribute to ESCC development by upregulating PRKAA1 and SQSTM1 as well as downregulating IL6. Citron kinase may serve as a promising therapeutic target for ESCC.

## Introduction

Esophageal cancer is a fatal malignant tumor affecting individuals worldwide (Bollschweiler et al., 2017; Huang and Yu, 2018). Global tumor epidemiological statistics show that there are nearly 572,000 new cases of esophageal cancer worldwide (Macedo-Silva et al., 2019). Esophageal squamous cell carcinoma (ESCC) is the main histological subtype of esophageal cancer, with a 5-year survival rate of less than 10% despite various treatments, such as surgery, chemotherapy, and radiotherapy (Zhong et al., 2019). Most importantly, approximately 50% of ESCC patients exhibit local infiltration and metastasis at the time of diagnosis, resulting in high mortality (Hirano and Kato, 2019; Minashi et al., 2019). Therefore, it is necessary to identify novel molecular targets for treatment of ESCC. To this end, further understanding of the relevant molecular mechanisms involved in ESCC development, such as analysis of the genetic landscape and intergenic interactions in ESCC, may provide new treatments and improve survival rate.

Citron kinase (CIT) is a serine/threonine kinase involved in the formation of mitotic intermediates (midbody) (D’Avino, 2017; Sahin et al., 2019). As an effector of Rho, CIT participates in cytokinesis by binding to Rho and regulates formation of mitotic intermediates through various protein components (El-Amine et al., 2019). In mammalian cells, depletion of CIT has been reported to cause failure of cytokinesis and promotes cell death in breast, cervical, and colorectal cancer cell lines (McKenzie and D’Avino, 2016). In addition, CIT has been associated with multiple tumors, such as breast cancer (Meng et al., 2019) and prostate cancer (Liu et al., 2020). Knockdown of CIT can inhibit the tumorigenic ability of SMMC-7221 cells *in vivo* by blocking cytokinesis (Fu et al., 2011). Furthermore, CIT is suggested as a potential target in anti-cancer therapy due to its role in chromosomal instability (CIN), which contributes to cancer progression, heterogeneity, and metastases as well as to drug resistance (Bassi et al., 2013). Taken together, these findings suggest that targeting CIT may serve as a potential anti-tumor treatment for a variety of cancers. However, there is no research on the role of CIT in ESCC.

In this study, to explore the promising tumorigenic effect of CIT in ESCC, we first examined CIT expression in ESCC tissues and several ESCC cell lines. We then examined the effect of CIT knockdown on ESCC cell proliferation and apoptosis *in vitro* as well as on tumor growth *in vivo*. To further delineate the possible molecular mechanism and regulatory network of CIT in esophageal cancer, microarray analysis was used to screen for CIT-regulated genes, followed by analysis of the signaling pathways by using Ingenuity Pathway Analysis (IPA). Furthermore, the expression of key CIT-regulated genes was validated using the clinical samples of ESCC. Our findings will provide deeper insight into the mechanisms involved in ESCC development and facilitate the design of promising approaches for the treatment of this disease.

## Materials and Methods

### Analysis of the Expression Level of CIT in ESCC Tissues According to Microarray Dataset GSE20347

The microarray dataset GSE20347, which contains the expression profile data of 17 pairs of ESCC tissues and adjacent normal tissues, was downloaded from the Gene Expression Omnibus database (GEO). The Affymetrix Human Genome U133A 2.0 Array was used. Expression of CIT in ESCC and normal tissues was then analyzed based on this dataset.

### Cell Culture and Transfection

The human ESCC cell lines EC9706, Eca-109, and TE-1 were obtained from the Type Culture Collection of the Chinese Academy of Sciences, Shanghai, China. They were cultured in Roswell Park Memorial Institute (RPMI) 1640 medium (Gibco, United States) containing 10% fetal bovine serum (FBS, Gibco, United States), 1% L-glutamine, and 1% penicillin/streptomycin (Hyclone, Shanghai, China). All cells were grown at 37°C in a humidified incubator with 5% CO_2_.

shRNA-CIT (shCIT) and shNRA-NC (shNC) were synthesized by Shanghai Genechem Co., Ltd. (Shanghai, China). The shCIT sequence was: 5′-GTCCTCATACCAGGATAAA-3′, and the shNC sequence was 5′-TTCTCCGAACGTGTCA CGT-3′. For the transfection, 1 × 10^5^ Eca-109 cells were plated into 6-well cell culture plates. Next day, cells were transfected with validated lentiviral constructs at an appropriate MOI. Infection efficiency was assessed with enhanced green fluorescent protein (EGFP) 72 h after infection.

### Real-Time Quantitative Reverse Transcription-PCR (qRT-PCR)

Total RNA was extracted with Trizol (Pufei, Shanghai, China) according to the manufacturer’s instructions. The extracted RNA (2 μg) was reverse-transcribed into cDNA using a reverse transcription kit (M-MLV Kit Promega, United States). qRT-PCR was performed with a qPCR Syber Green kit (SYBR Master Mix, Takara, Beijing, China) on an ABI 7500 Real-Time PCR System (Applied Biosystems, Foster City, CA). Primer 6.0^1^ was used to design primers. The following primers were used: CIT, 5′-CAGGCAAGATTGAGAACG-3′ (forward) and 5′-GCACGATTGAGACAGGGA-3′ (reverse); GAPDH, 5′-TGACTTCAACAGCGACACCCA-3′ (forward) and 5′-CACCCTGTTGCTGTAGCCAAA-3′ (reverse). GAPDH was used for normalization. The relative gene expression was calculated using the 2^–ΔΔCT^ method as previously described (Livak and Schmittgen, 2001).

### Western Blot

After treatment, the cell lysate was prepared with RIPA Buffer (Beyotime, Shanghai, China), followed by determination of total protein concentration using the BCA Protein Assay Kit (Beyotime, Shanghai, China). Then, the protein extract (30 μg) was mixed with loading buffer, heated at 100°C for 5 min, separated by SDS-PAGE electrophoresis, and transferred to a polyvinylidene fluoride (PVDF) membrane (Millipore, United States). After blocking with TBST solution containing 5% skimmed milk, primary antibodies were added overnight at 4°C. Detailed information on the primary antibodies used is provided in Table 1. After probing with secondary antibody (goat anti-mouse IgG, 1:5000, Santa Cruz) conjugated with horseradish peroxidase, membranes were developed with enhanced chemiluminescence reagent ECL (PE0010, Solarbio, China). GAPDH served as an internal normalization control. Quantification of the relative protein level was performed through gray level by using Quantity-One software (NIH).

**TABLE 1:** Information of primary antibodies used in western blot.

Name	Source	Brand	Dilution ratio
ACACA	Rabbit	CST	1:1000
SDC2	Rabbit	ABCAM	0.1–0.5 ug/ml
IL6	Rabbit	ABCAM	1:500–1:2000
PRKAA1	Rabbit	CST	1:1000
SQSTM1	Mouse	CST	1:1000
GAPDH	Mouse	SANTA CRUZ	1:2000

### Cell Proliferation

For the proliferation assay, 5,000 Eca-109 cells transfected with LV-shCIT and LV-shNC were plated in 96-well plates, and the number of metabolically active cells at days 1, 2, 3, 4, and 5 was counted with Celigo image cytometry (Nexcelom, United States) (Chan et al., 2016).

In the meantime, MTT assay was performed to evaluate cell proliferation from the perspective of cell viability. Cells were plated in 96-well plates until they were fully attached. The 20 μL MTT reagent (5 mg/mL, Sigma, United States) was added and the plate was incubated for another 4 h. Finally, 100 μL of DMSO was used to dissolve the precipitate. The absorbance was measured at 490 nm using a microplate reader (Tecan infinite, United States).

### Colony Formation Assay

Eca-109 cells transfected with LV-shCIT and LV-shNC (800 cells per well) were plated in 6-well plates and then cultured for 14 days. The culture medium was changed every 3 days. On the 14^th^ day, cells were fixed with 4% paraformaldehyde for 30–60 min. After rinsing with PBS, cells were stained with crystal violet staining solution for 10–20 min. Cells were rinsed with ddH_2_O several times, colonies were counted, and images were acquired.

### Cell Apoptosis

After being transfected with LV-shCIT or LV-shNC for 3 days, Eca-109 cells were washed with pre-cooled PBS and then resuspended in binding buffer. Cells were stained with Annexin V Apoptosis Detection Kit (Invitrogen, China) in the dark and incubated at room temperature for 15 min. After rinsing the cells, cell apoptosis was detected with flow cytometry (Millipore, United States).

### Gene Microarray

Microarray analysis of Eca-109 cells transfected with LV-shCIT or LV-shNC was conducted. Briefly, total RNA was initially isolated from Eca-109 cells using TRIzol, reverse-transcribed, and labeled with biotin using the GeneChip^®^ 3′ IVT Express kit. Labeled cDNA was then hybridized onto the GeneChip^®^ PrimeView^*TM*^ Human Gene Expression Array overnight at 60°C. Chips were then treated with GeneChip^®^Hybridization Wash and Stain kit and scanned directly post-hybridization using a GeneChip^®^ Scanner 3000. All GeneChip^®^ products were obtained from Affymetrix (Thermo Fisher Scientific, Inc., Waltham, MA, United States). The microarray data was deposited into GEO with the accession number of GSE161804^2^.

Using GeneSpring (Version 11; Agilent Technologies, Inc.), microarray data were analyzed and normalized with the GeneSpring normalization algorithm. Finally, genes with fold changes >1.5, and *P* ≤ 0.05 between LV-shCIT and LV-shNC-transfected cells were identified as differentially expressed genes (DEGs).

### Ingenuity Pathway Analysis (IPA)

Differentially expressed genes (DEGs) were imported into the IPA tool^3^ (Ingenuity^®^ Systems, Redwood City, CA, United States). The differentially expressed data were interpreted using the “core analysis” function, which included “Disease and Functions,” “pathway,” and “Molecular Network.” Differentially expressed genes were introduced into the IPA database and then ranked based on P-value to measure enrichment of DEGs in the present dataset. Then, the z-score algorithm of IPA software was used to predict the regulation effect (upregulation or downregulation of the pathway). Ingenuity Pathway Analysis analyses included annotation of biological networks of the dataset of interest and its global functions, functional signaling pathways, and downstream targets.

### *In vivo* Experiments

All procedures were performed according to the protocols approved by the Institutional Animal Ethics Care and Use Committee of Shanghai University of Traditional Chinese Medicine. The 4-week-old female BALB/c nude mice were purchased from Shanghai Ling Chang Biological Technology Co., Ltd. and were housed in sterilized cages (4 animals/cage). To establish a nude mouse xenograft model, 4 × 10^6^ Eca-109 cells transfected with LV-shCIT or LV-shNC were subcutaneously injected into the right flank of nude mice. Tumor diameters were measured twice or thrice every week, and tumor volumes were calculated using the following formula:

$$\text{V}\,\left({\text{mm}}^{3}\right)=\pi /6\times \text{L}\times \text{W}\times \text{W}\left(L:\mathrm{long}\,\mathrm{diameter};W:\mathrm{short}\,\mathrm{diameter}\right).$$

All mice were sacrificed on day 23.

### Clinical Validation of the Expression of PRKAA1, SQSTM1, and IL6 by qRT-PCR

To confirm the results of IPA analysis, we detected the expression of targets of CIT, including PRKAA1, SQSTM1, and IL6 in clinical samples of ESCC by qRT-PCR. In total, 10 pairs of ESCC tissues and adjacent normal tissues were obtained from 10 ESCC patients who were recruited in the Shanghai Chest Hospital. All diagnoses were based on histopathological evidence. These tissues after surgical separation were immediately frozen in liquid nitrogen and stored at −80°C until use. This study was approved by the Ethics Committee of Shanghai Chest Hospital [No. KS (Y) 1943]. All patients were notified consent for the use of their tissues for research.

RNA extraction and qRT-PCR assay were conducted as mentioned above. The following primers were used: actin, 5′-GGACTTCGAGCAAGAGATGG-3′ (forward) and 5′- AGCACT GTGTTGGCGTACAG-3′ (reverse); PRKAA1, 5′-TGCAGGC CCAGAGGTAGATA-3′ (forward) and 5′-ATTGTGGCCCTCT TCATGGG-3′ (reverse); SQSTM1, 5′-GCCTGTCCCTGAAAG AGAAGA-3′ (forward) and 5′-TGCTTTGTCTTGCTAACCC TCA-3′ (reverse); and IL6, 5′-TGCAATAACCACCCCTGACC-3′ (forward) and 5′-GTGCCCATGCTACATTTGCC-3′ (reverse).

### Statistical Analysis

All data were expressed as the mean ± standard deviation, and comparisons between two groups were evaluated via Student’s *t*-test using SPSS 23.0 (SPSS Inc., Chicago, Illinois, United States). *P* < 0.05 was considered statistically significant.

## Results

### High Expression of CIT in ESCC Tissues and Cell Lines

**FIGURE 1:**
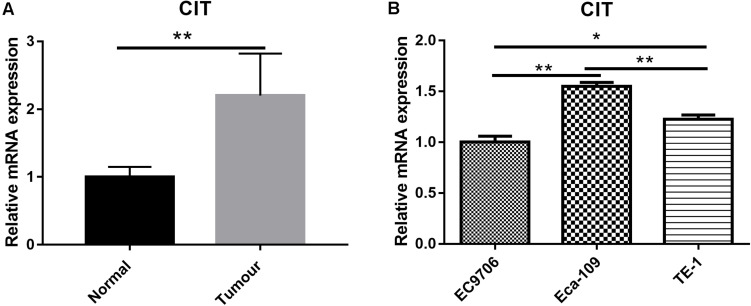
Expression of CIT in ESCC tissues and cell lines. **(A)** The gene expression profile of CIT in ESCC tissues and adjacent normal tissues based on microarray data GSE20347. Expression of CIT is significantly higher in ESCC tissues than that in normal tissues adjacent to tumor tissues. **(B)** The level of CIT mRNA in ESCC cell lines (EC9706, Eca-109, and TE-1) was identified by qRT-PCR and analyzed by 2^–ΔΔCT^ method. * *P* < 0.05 and ** *P* < 0.01.

To study the expression profile of CIT in ESCC, we downloaded the mRNA expression profile (ID: GSE20347) of ESCC from GSE20347 and found that the mRNA expression level of CIT was significantly higher in ESCC tissues than that in adjacent non-tumor tissues (FC = 2.14). Among the 17 samples, nine samples showed upregulated expression of CIT, eight showed unchanged expression, and none showed downregulation (Figure 1A).

We further examined CIT expression with qRT-PCR in a series of ESCC cell lines, including Eca-109, EC9706, and TE-1. Among the three ESCC cell lines, the Eca-109 cell line showed the highest expression level of CIT (Figure 1B), and it was chosen for subsequent experiments.

### Knockdown of CIT Inhibited Eca-109 Cell Proliferation and Promoted Apoptosis

**FIGURE 2:**
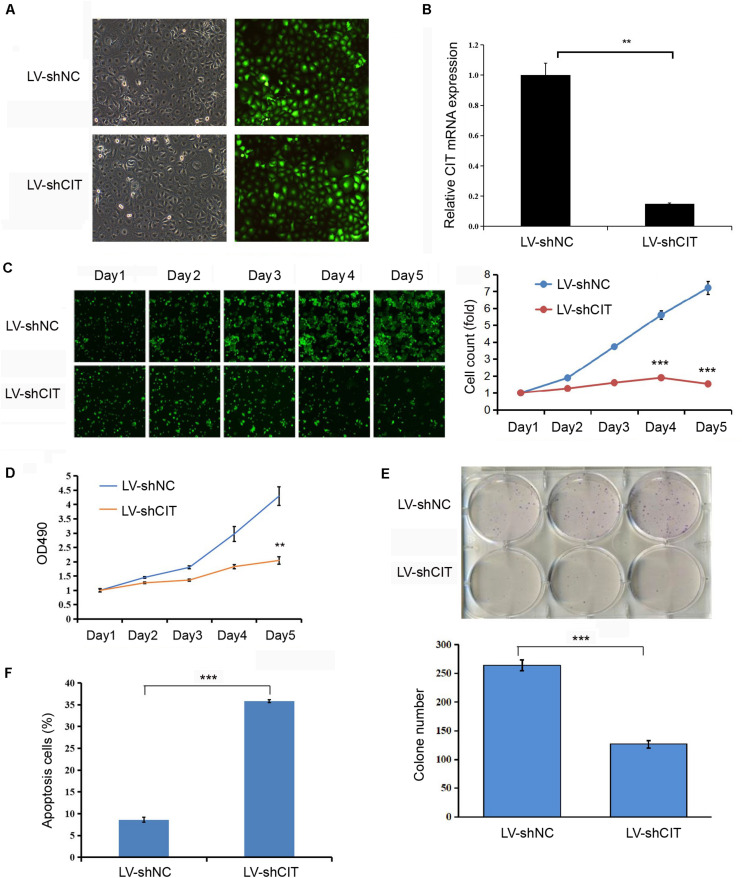
Effect of knockdown of CIT on Eca-109 cell proliferation and apoptosis *in vitro*. **(A)** Infection of Eca-109 cells with LV-shCIT or LV-shNC. The efficiency of transfection was monitored with co-expression of EGFP. **(B)** The relative expression level of CIT mRNA in LV-shCIT or LV-shNC transfected Eca-109 cells. **(C)** Images and Celigo cell count of fluorescent LV-shCIT or LV-shNC-transfected Eca-109 cells 5 days after transfection. **(D)** MTT assay was conducted to record the fold change in absorbance at 490 nm within 5 days after transfection. **(E)** Colony formation assay of LV-shCIT or LV-shNC Eca-109 cells. **(F)** Flow cytometry histograms showing the percentage of apoptotic cells after transfection. ** *P* < 0.01 and *** *P* < 0.001.

CIT was successfully knocked down by transfecting LV-shCIT in Eca-109 cells, and transfection efficiency was verified by both EGFP fluorescence (Figure 2A) and qRT-PCR (*P* < 0.01, Figure 2B). After LV-shRNA infection, the expression of CIT in Eca-109 cells of the LV-shCIT group was significantly reduced (*P* < 0.01), and the knockdown efficiency reached 85.1% (Figure 2B). To explore the effect of depletion of CIT on Eca-109 cell proliferation, we performed Celigo cell counting (Figure 2C), MTT assay (Figure 2D), and colony formation assay (Figure 2E). Consistent results of the three methods showed that proliferation of LV-shCIT-transfected Eca-109 cells was significantly inhibited compared with that of LV-shNC-transfected cells (all *P* < 0.01, Figures 2C–E). Furthermore, it was also essential to confirm whether CIT acted as an anti-apoptosis factor in ESCC cells. To this end, we performed flow cytometry assays to detect Eca-109 cell apoptosis. Apoptosis of LV-shCIT-transfected Eca-109 cells was obviously increased at 72 h after transfection, and the ratio of apoptotic cells was nearly 5 times higher in LV-shCIT-transfected cells than that in LV-shNC-transfected cells (*P* < 0.001, Figure 2F).

### Downregulation of CIT Reduced Tumorigenicity *in vivo*

**FIGURE 3:**
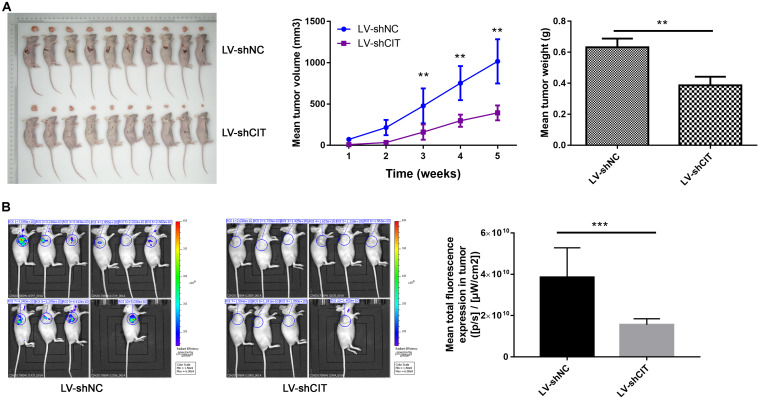
Effects of CIT knockdown on tumor growth *in vivo*. **(A)** Changes in tumor volume and tumor weight in nude mice with LV-shCIT or LV-shNC xenografts. Tumor was smaller in LV-shCIT nude mice than that in LV-shNC mice. **(B)** Tumor fluorescence imaging of the two groups of nude mice (left). Mean total fluorescence expression in tumors of nude mice in LV-shCIT or LV-shNC groups(right); fluorescence intensity was significantly higher in nude mice in LV-shCIT group than that in LV-shNC group. ** *P* < 0.01 and *** *P* < 0.001.

Considering the effect of CIT knockdown on the suppression of cell proliferation and the promotion of apoptosis *in vitro*, we investigated whether reduced CIT levels suppressed tumorigenicity in nude mice with Eca-109 xenografts. To this end, Eca-109 cells transfected with LV-shCIT or LV-shNC were injected subcutaneously into nude mice. Tumor volume was measured 2–3 times per week. After 23 days, all mice were sacrificed, and tumor tissues were removed surgically. The volume and weight of tumors were significantly decreased in LV-shCIT xenografts compared to those in LV-shNC xenografts (*P* < 0.01, Figure 3A). At the same time, fluorescence imaging showed that mice in the LV-shCIT group exhibited a smaller fluorescence-labeled area than mice in the LV-shNC group (*P* < 0.001, Figure 3B). These data indicated that downregulation of CIT remarkably suppressed tumorigenicity *in vivo*.

### Downregulation of CIT Altered Gene Expression Profiles in ESCC Cells

**FIGURE 4:**
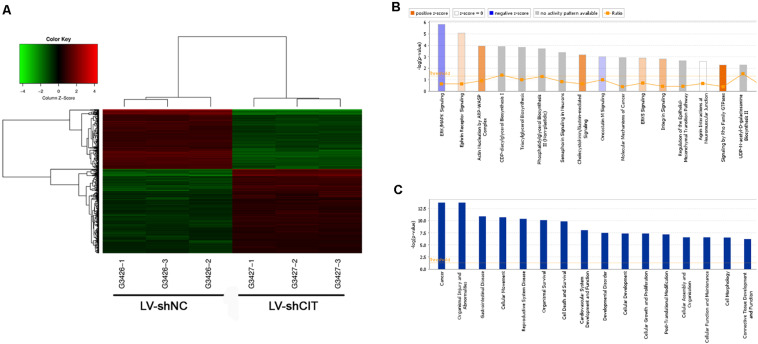
Heatmap and enrichment analysis of differentially expressed genes (DEGs). **(A)** Heatmap showing DEGs from all samples. Red indicates that gene expression was relatively upregulated, green indicates downregulation, black suggests no change, and gray suggests that gene signal value was not detected. **(B)** Enrichment analysis of DEGs in classical signaling pathways. All pathways were ranked by –Log (P-value). Orange represents Z-score > 0, and blue represents Z-score < 0. Z-score > 2 represents pathways that were significantly activated, and Z-score < −2 represents pathways that were significantly suppressed. Ratio indicates the ratio of the number of DEGs to all genes in the signaling pathway. **(C)** Enrichment analysis of DEGs in the classification of diseases and functions. All diseases and functions were ranked by –Log (P-value).

To further investigate the possible molecular mechanism of CIT function in ESCC, microarray analysis was used to identify DEGs between LV-shCIT group and the LV-shNC group. Our results showed that there were 805 DEGs (466 upregulated and 339 downregulated) between the two groups. Citron kinase expression was reduced by approximately 2.2 times in LV-shCIT-transfected cells. The heat map of DEGs is shown in Figure 4A. Citron kinase enrichment analysis based on all DEGs showed that signaling by Rho family GTPases, a typical cancer-related pathway, was remarkably activated by CIT, with a Z-score of 2.138 (Figure 4B). The disease and functional enrichment analysis based on the DEGs indicated that cell migration (*Z* = 3.377) and cell movement (*Z* = 3.332) of tumor cell lines were activated by CIT (Figure 4C).

### The Genetic Interaction and Hypothetical Regulatory Network of CIT

**FIGURE 5:**
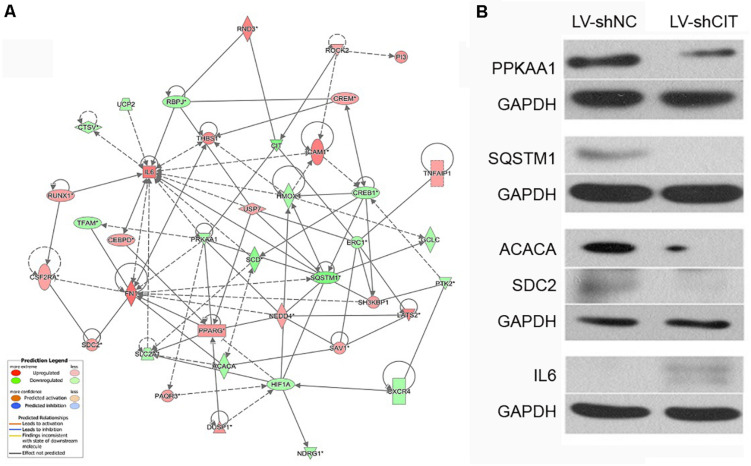
CIT-related genetic network and downstream gene verification. **(A)** Gene interaction network showing the interaction network between molecules related to cell proliferation and apoptosis. **(B)** Expression of several downstream key genes (PRKAA1, SQSTM1, ACACA, SDC6, and IL6) was verified with western blot analysis.

According to our IPA analysis, genes related to cell proliferation and apoptosis were found to generate a CIT-based genetic network (Figure 5A). The results showed that CIT was likely to affect cell proliferation and apoptosis by affecting expression of key genes such as protein kinase AMP-activated catalytic subunit alpha 1 (PRKAA1), sequestosome 1 (SQSTM1), acetyl-CoA carboxylase alpha (ACACA), syndecan 2 (SDC2), and interleukin 6 (IL6). Next, we used western blot to verify that CIT regulated protein expression of these genes. Compared with the LV-shNC group, the protein expression levels of PRKAA1 and SQSTM1 in the LV-shCIT group were significantly reduced, whereas IL6 protein expression was significantly increased (Figure 5B).

### Clinical Validation of the Expression of PRKAA1, SQSTM1, and IL6 by qRT-PCR

**FIGURE 6:**
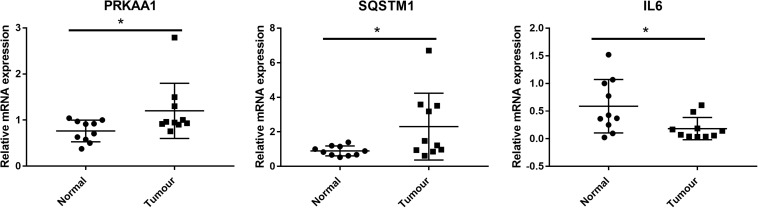
Clinical validation of the expression of CIT downstream genes (including PRKAA1, SQSTM1, and IL6) by qRT-PCR. PRKAA1 and SQSTM1 expression were significantly upregulated in ESCC tissues in comparison with adjacent normal tissues, while the IL6 expression was remarkably downregulated. * *P* < 0.05.

We further detected whether the targets of CIT including PRKAA1, SQSTM1, and IL6 were dysregulated in clinical samples of ESCC by qRT-PCR. As results, the expression levels of PRKAA1 and SQSTM1 were significantly upregulated in ESCC tissues in comparison with adjacent normal tissues, while the IL6 expression was remarkably downregulated (P < 0.05, Figure 6).

## Discussion

Esophageal cancer is a common malignant tumor of the digestive system. ESCC is a subtype of esophageal cancer and characterized by advanced diagnosis and high mortality. Although progress has been made in surgical and non-surgical treatments in the past few decades, prognosis of ESCC remains poor due to the lack of appropriate biomarkers for early diagnosis and effective treatment (Kato and Nakajima, 2013; Watanabe et al., 2020). Our study found that CIT expression was increased in ESCC tissues. Knockdown of CIT significantly suppressed Eca-109 cell proliferation and promoted cell apoptosis *in vitro*. In addition, knockdown of CIT significantly reduced tumorigenicity of Eca-109 cells *in vivo*. These data suggest a key role of CIT in ESCC.

Citron kinase (CIT) is an important kinase in cell division and has been considered to function only during cytokinesis (Li et al., 2016). Some recent studies have found that CIT is essential for several cancers. Meng et al. (2019) found that knockdown of CIT inhibited aggressiveness and tumorigenesis of breast cancer cells. Moreover, Sahin et al. (2019) confirmed that CIT is enhanced in multiple myeloma, and silencing of CIT inhibited the growth of multiple myeloma *in vivo* and *in vitro*. In this study, CIT was highly expressed in both ESCC tissues and cell lines. Knockdown of CIT markedly inhibited proliferation and promoted cell apoptosis in Eca-109 cells *in vitro*. Moreover, *in vivo* experiments also confirmed that knocking down CIT inhibited tumorigenicity of Eca-109 cells. These findings reveal the oncogenic role of CIT in ESCC.

As ESCC is a complex disease caused by multiple interacting genetic mechanisms, the regulatory mechanism of CIT in the pathogenesis of ESCC is unclear. Previous studies have found that p27 acts as a tumor suppressor by closely binding to CIT to prevent its interaction with its activator RhoA, thereby regulating cytokinesis (Serres et al., 2012). Recently, a study has found that inactivation of CIT significantly reduces RAD51, a factor that helps to repair DNA damage, thus causing accumulation of DNA damage in medulloblastoma cells and finally leading to apoptosis (Pallavicini et al., 2020). In the current study, the microarray results revealed several key regulators that may be controlled by CIT. Among these genes, PRKAA1, SQSTM1, and IL6 are closely related to cell proliferation and apoptosis. PRKAA1 encodes for the adenosine monophosphate activated protein kinase (AMPK), which regulates tumor growth and proliferation by regulating energy metabolism (Dargiene et al., 2018). He et al. pointed out that LINC00473 regulated ESCC development through the miR-497-5p/PRKAA1 axis (He, 2019). SQSTM1, also known as p62, plays an important role in autophagy regulation, proteasome pathway, NF-κB, apoptosis, and other signaling pathways (Zhang et al., 2019). It is highly expressed in prostate cancer, breast cancer, and lung cancer, and promotes tumor cell proliferation (Falasca et al., 2015; Li et al., 2017; Yang et al., 2019). SQSTM1 has also been shown to protect ESCC cells from apoptosis by stabilizing SKP2 under serum starvation conditions (Shi et al., 2018). IL6 is an important inflammatory cytokine that has various functions in immune cell responses and tumor growth regulation. Studies have found that IL6 can promote proliferation, invasion, and metastasis of Eca-109 cells by activating the JAK/STAT signaling pathway (Xu et al., 2020). A preliminary protein chip study suggested that IL6 levels can be used as a marker for the diagnosis of ESCC at an early stage (Tong et al., 2018). Here, we found that knockdown of CIT resulted in a remarkable decrease in protein expression levels of PRKAA1 and SQSTM1 as well as an obvious increase in IL6 protein expression. Importantly, the expression levels of PRKAA1 and SQSTM1 were significantly upregulated in clinical ESCC tissues while the IL6 expression was remarkably downregulated. Given the key role of PRKAA1, SQSTM1, and IL6 in ESCC, we speculate that CIT may play a key role in ESCC by regulating expression of these proteins.

However, our study has some limitations. Firstly, there was no data on clinical characteristics and prognosis, thus, the clinical significance of CIT should be further explored. Secondly, only one cell line was used to investigate the role of CIT knockdown *in vitro*. The use of more ESCC cell lines to investigate the role CIT knockdown may provide additional evidence to support our findings. Thirdly, the possible molecular mechanisms of CIT were analyzed based on our bioinformatics analysis without systematic experimental verification. More *in vivo* studies are required to confirm our hypothesis.

In summary, our findings indicate that CIT is increased in ESCC. Citron kinase may function as an oncogene in ESCC by upregulating PRKAA1 and SQSTM1 as well as downregulating IL6. Citron kinase may serve as a biomarker for early diagnosis and a therapeutic target for esophageal cancer.

## Data Availability Statement

The microarray data was deposited into GEO with the accession number of GSE161804 (https://www.ncbi.nlm.nih.gov/geo/query/acc.cgi?acc=GSE161804).

## Ethics Statement

This study was approved by the Ethics Committee of Shanghai Chest Hospital [No. KS(Y) 1943]. All procedures of animal study were performed according to the protocols approved by the Institutional Animal Ethics Care and Use Committee of Shanghai University of Traditional Chinese Medicine.

## Author Contributions

WL and YD conceived and designed the project and collected the data. QC, YW, and XY performed the interpretation of data and statistical analysis. WL wrote the manuscript. XC revised the manuscript. All authors read and approved the final manuscript.

## Conflict of Interest

The authors declare that the research was conducted in the absence of any commercial or financial relationships that could be construed as a potential conflict of interest.

## Footnotes

1 http://www.premierbiosoft.com/

2 https://www.ncbi.nlm.nih.gov/geo/query/acc.cgi?acc=GSE161804

3 http://www.ingenuity.com
